# Deep vein thrombosis and coagulopathy following cyclone events: pathophysiology, risk factors, and prevention

**DOI:** 10.1097/MS9.0000000000004898

**Published:** 2026-03-25

**Authors:** Emmanuel Ifeanyi Obeagu, Ezeldine Abdalhabib

**Affiliations:** a Division of Haematology, Department of Biomedical and Laboratory Science, Africa University, Mutare, Zimbabwe; b Department of Molecular Medicine and Haematology, School of Pathology, Faculty of Health Sciences, University of the Witwatersrand, Johannesburg, South Africa; c Department of Clinical Laboratory Sciences, College of Applied Medical Sciences, Jouf University, AlQurayyat, Saudi Arabia

**Keywords:** coagulopathy, cyclone, deep vein thrombosis, disaster health, thrombosis risk

## Abstract

Cyclone events, characterized by high winds, flooding, and mass displacement, pose significant challenges to public health infrastructure and individual well-being. While acute injuries and infectious diseases are the primary focus of disaster response, thromboembolic complications such as deep vein thrombosis (DVT) and coagulopathies remain underrecognized. The convergence of immobility, dehydration, trauma, and systemic inflammation during and after cyclones creates an ideal environment for thrombus formation, particularly among vulnerable populations. This review explores the pathophysiological mechanisms linking cyclone-related stressors to DVT and coagulation disorders, emphasizing the roles of Virchow’s triad, immune activation, and disaster-induced comorbid conditions. Specific risk factors – including advanced age, pregnancy, chronic disease, and restricted mobility in shelters – are discussed in detail. We also highlight the potential for sepsis-induced disseminated intravascular coagulation (DIC) following infectious outbreaks in cyclone-affected regions. Effective prevention and management strategies are urgently needed in disaster-prone settings. These include early mobilization, hydration, use of compression stockings, pharmacologic thromboprophylaxis, and the integration of thrombotic risk screening into disaster response protocols. Improved awareness, targeted interventions, and further research can help reduce the burden of cyclone-associated thromboembolic events and improve outcomes in affected populations.

## Introduction

Cyclones, also known as hurricanes or typhoons depending on their geographical location, are among the most devastating natural disasters worldwide. They cause widespread destruction through high winds, flooding, and infrastructural damage, severely impacting communities and health care systems. Beyond the immediate trauma and injuries, cyclones often precipitate secondary health complications that contribute significantly to morbidity and mortality. One such set of complications involves thrombotic disorders, specifically deep vein thrombosis (DVT) and coagulopathy, which have increasingly been recognized as critical post-disaster health hazards^[^1^]^. DVT refers to the formation of blood clots within the deep veins, commonly in the lower limbs, which can lead to life-threatening pulmonary embolism (PE) if left untreated. Coagulopathy, on the other hand, is a broader condition characterized by abnormal blood clotting, which can manifest as either excessive clotting or bleeding. The emergence of these conditions following cyclones poses unique clinical challenges due to disrupted health care infrastructure, delayed diagnosis, and limited access to preventive and therapeutic resources^[^2^]^.

HIGHLIGHTS

- Cyclones create prothrombotic conditions through immobility, dehydration, trauma, and inflammation.
- Disrupted health care and anticoagulation access amplify venous thromboembolism risk.
- Endothelial dysfunction and cytokine activation drive post-disaster coagulopathy.
- Deep vein thrombosis remains underdiagnosed due to surveillance and diagnostic gaps.
- Mobility, hydration, and risk-based prophylaxis reduce preventable thrombotic deaths.

The pathogenesis of DVT and coagulopathy in the aftermath of cyclone events is multifactorial. Key contributors include prolonged immobility due to injury or shelter confinement, dehydration from limited water availability, and physiological stress responses. The interplay between venous stasis, endothelial injury, and hypercoagulability – components of Virchow’s triad – creates an ideal environment for thrombogenesis. Furthermore, systemic inflammation and trauma-induced endothelial dysfunction exacerbate coagulation abnormalities, sometimes resulting in severe complications such as DIC^[^3^]^. Risk factors for thrombotic complications in cyclone-affected populations extend beyond physiological changes. Environmental factors such as heat, humidity, and poor sanitary conditions amplify the risk of dehydration and infection, which further stimulate coagulation cascades. Psychological stress and nutritional deficiencies also play contributory roles. Pre-existing conditions like obesity, cardiovascular disease, and inherited thrombophilias increase individual susceptibility, complicating the clinical picture^[^4,5^]^.

The post-cyclone health care environment often suffers significant disruption, hampering timely diagnosis and management of thrombotic disorders. Medical facilities may be damaged or overwhelmed, diagnostic tools such as Doppler ultrasound may be unavailable, and essential medications including anticoagulants can be scarce. These limitations necessitate prioritizing prevention and early detection strategies in disaster response planning to mitigate adverse outcomes^[^6,7^]^. Preventive measures are critical in managing thrombotic risks after cyclones. Early mobilization, hydration maintenance, and pharmacological prophylaxis with anticoagulants have demonstrated efficacy in reducing DVT incidence in vulnerable groups. However, balancing bleeding risks and resource availability requires careful clinical judgment, especially in disaster settings. Training health care workers and disaster responders to recognize and address thrombosis symptoms can facilitate timely intervention^[^8,9^]^.

## Aim

This narrative review aims to provide a comprehensive overview of the pathophysiological mechanisms, specific risk factors, and effective prevention and management strategies related to DVT and coagulopathy following cyclone events.

## Methods

This narrative review was conducted to synthesize existing evidence on DVT and coagulopathy following cyclone events, with emphasis on pathophysiology, risk factors, and prevention strategies. A comprehensive literature search was undertaken using PubMed, Scopus, Web of Science, and Google Scholar, covering publications from database inception to the most recent available literature. Search terms included combinations of keywords and Medical Subject Headings related to cyclones, hurricanes, tropical storms, natural disasters, DVT, venous thromboembolism, coagulopathy, endothelial dysfunction, dehydration, immobility, inflammation, and disaster medicine. Reference lists of selected articles were also screened to identify additional relevant studies.

Eligible sources comprised original research articles, observational studies, systematic and narrative reviews, clinical guidelines, and reports from international health and disaster-response organizations. Case reports and small case series were included where higher-level evidence was limited, reflecting the emerging nature of cyclone-related thrombotic research. Studies focusing on other natural disasters were considered when mechanistic or clinical findings were applicable to cyclone settings. Article selection was based on relevance to post-cyclone thrombotic risk, biological mechanisms, clinical manifestations, or preventive strategies. Data extraction emphasized study context, population characteristics, thrombotic outcomes, proposed pathophysiological pathways, identified risk factors, and mitigation approaches. Owing to heterogeneity in study design and outcomes, findings were synthesized qualitatively without meta-analysis. This review followed established principles for narrative reviews and aimed to provide a clinically and public health–relevant synthesis of the literature. Ethical approval was not required as the study involved analysis of previously published data.

## Pathophysiology of DVT and coagulopathy in cyclone settings

The pathophysiology of DVT and coagulopathy following cyclone events is complex and multifactorial, involving a convergence of environmental, physiological, and biochemical factors that collectively promote thrombus formation and coagulation disturbances. Understanding these mechanisms is essential for identifying at-risk individuals and developing targeted interventions^[^3^]^. Central to thrombogenesis is Virchow’s triad, which describes three primary factors contributing to venous thrombus formation: venous stasis, endothelial injury, and hypercoagulability. Post-cyclone conditions favor all three components. Immobilization is common due to injuries, displacement to shelters, or limited mobility caused by damaged infrastructure, leading to venous stasis in the lower extremities. Stagnant blood flow increases the likelihood of clot formation by allowing platelets and clotting factors to accumulate and activate^[^3^]^.

Endothelial injury plays a critical role in activating the coagulation cascade. Physical trauma sustained during cyclones can directly damage vascular endothelium, exposing subendothelial collagen and tissue factor that trigger platelet adhesion and activation of the extrinsic coagulation pathway. Additionally, systemic inflammatory responses to trauma, infection, and stress promote endothelial dysfunction by releasing pro-inflammatory cytokines such as tumor necrosis factor-alpha (TNF-α) and interleukins, which upregulate adhesion molecules and procoagulant factors on endothelial cells^[^10^]^. Hypercoagulability in post-cyclone survivors is influenced by dehydration and hemoconcentration due to inadequate fluid intake and heat exposure. Dehydration increases blood viscosity and concentrates clotting factors, tipping the balance toward a prothrombotic state. Moreover, stress-induced release of catecholamines and cortisol enhances platelet reactivity and coagulation factor synthesis. Inflammatory mediators further amplify coagulation by disrupting the anticoagulant pathways, including antithrombin and protein C systems, creating a systemic milieu favorable for thrombus propagation^[^11^]^.

Coagulopathy following cyclone-related trauma may manifest as DIC, a severe condition characterized by widespread activation of clotting cascades leading to microvascular thrombosis and consumption of clotting factors. This paradoxical state results in simultaneous risk of bleeding and thrombosis, complicating clinical management. DIC often occurs in critically ill individuals with severe injuries, sepsis, or extensive tissue damage commonly observed in disaster scenarios (Table 1)^[^12^]^.

**Table 1:** Pathophysiology of DVT and coagulopathy in cyclone settings.

Pathophysiological domain	Cyclone-related trigger	Biological mechanism	Thrombotic consequence
Venous stasis	Prolonged immobility in shelters, overcrowding, restricted movement	Reduced venous return and shear stress leading to coagulation cascade activation	Lower-limb deep vein thrombosis
Dehydration and hemoconcentration	Disrupted water supply, heat exposure, limited access to fluids	Increased blood viscosity, platelet aggregation, reduced plasma volume	Hypercoagulability and venous thrombosis
Endothelial injury	Trauma from debris, flooding injuries, crush injuries	Exposure of tissue factor and subendothelial collagen, endothelial activation	Initiation of clot formation
Systemic inflammation	Infections, tissue injury, psychological stress	Cytokine-mediated upregulation of tissue factor and suppression of fibrinolysis	Sustained prothrombotic state
Endothelial dysfunction	Oxidative stress, inflammation, hypoxia	Reduced nitric oxide bioavailability and impaired antithrombotic signaling	Enhanced platelet adhesion and thrombosis
Trauma-induced coagulopathy	Major physical injury, hypoperfusion, acidosis	Dysregulated coagulation with transition from hypocoagulable to hypercoagulable states	Combined bleeding risk and delayed thrombosis
Disruption of anticoagulation therapy	Loss of medications, health care access interruption	Rebound hypercoagulability following abrupt anticoagulant cessation	Recurrent or new venous thromboembolism
Reduced fibrinolysis	Inflammatory and stress hormone activation	Increased plasminogen activator inhibitor activity	Persistence and propagation of thrombi

## Risk factors specific to post-cyclone environments

Post-cyclone environments create a unique constellation of risk factors that significantly increase the likelihood of developing DVT and coagulopathy. These factors stem from both the physical consequences of the disaster and the ensuing socio-environmental conditions that collectively predispose affected individuals to thrombotic complications (Table 2)^[^13^]^.

**Table 2:** Risk factors specific to post-cyclone environments.

Risk factor category	Specific risk factor	Post-cyclone context	Impact on thrombotic risk
Demographic factors	Advanced age	Older adults disproportionately displaced and immobilized	Increased baseline venous thromboembolism risk
Physiological states	Pregnancy and postpartum period	Limited antenatal/postnatal care, dehydration, immobility	Heightened hypercoagulability
Pre-existing conditions	Prior DVT or pulmonary embolism	Interrupted follow-up and anticoagulation	High risk of recurrence
Pre-existing conditions	Malignancy	Treatment disruption and systemic inflammation	Sustained prothrombotic state
Mobility-related factors	Prolonged immobility	Overcrowded shelters, fear of movement, lack of transport	Venous stasis and clot formation
Environmental exposure	Dehydration	Disrupted water supply, heat stress	Hemoconcentration and increased viscosity
Environmental exposure	High ambient temperature	Post-cyclone heat waves, poor shelter ventilation	Exacerbation of dehydration and stasis
Trauma-related factors	Soft tissue and crush injuries	Debris-related trauma during and after cyclones	Endothelial injury and coagulation activation
Infection-related factors	Acute and chronic infections	Wound infections, waterborne diseases	Inflammatory-mediated hypercoagulability
Healthcare disruption	Loss of anticoagulant therapy	Medication shortages, facility damage	Rebound hypercoagulability
Healthcare disruption	Delayed diagnosis	Limited imaging and laboratory capacity	Progression to advanced DVT or PE
Social determinants	Poverty and displacement	Prolonged shelter stays, poor nutrition	Compounded thrombotic vulnerability

### Prolonged immobilization

One of the most prominent risk factors is prolonged immobility resulting from cyclone-induced injuries, displacement to emergency shelters, or infrastructural damage limiting movement. Victims may remain confined in cramped, crowded spaces with limited ability to change position for extended periods, leading to venous stasis in the lower limbs. Immobilization impairs the muscle pump mechanism crucial for venous return, thereby facilitating clot formation^[^14^]^.

### Dehydration and fluid imbalance

Cyclone aftermath often involves scarcity of clean water and inadequate hydration, compounded by hot and humid weather conditions common in many cyclone-prone regions. Dehydration increases blood viscosity and hemoconcentration, enhancing the hypercoagulable state. This fluid imbalance exacerbates vascular endothelial stress and promotes platelet aggregation, both of which contribute to thrombogenesis^[^15^]^.

### Trauma and tissue injury

Physical injuries sustained during cyclones – ranging from fractures to soft tissue damage – trigger inflammatory responses that amplify coagulation activity. Endothelial disruption caused by trauma exposes tissue factor, initiating coagulation cascades. Additionally, trauma-induced systemic inflammation releases pro-inflammatory cytokines that enhance coagulation and impair natural anticoagulant pathways, increasing the risk of both localized and disseminated thrombotic events^[^16^]^.

### Environmental and psychological stressors

The chaotic and often unsanitary conditions in post-cyclone settings, including overcrowding, exposure to pathogens, and poor nutrition, contribute to systemic stress that affects hemostatic balance. Psychological stress activates the hypothalamic-pituitary-adrenal axis, leading to increased catecholamine and cortisol levels, which can promote platelet activation and hypercoagulability. Furthermore, infections common in disaster settings may precipitate sepsis-induced coagulopathy^[^17^]^.

### Pre-existing medical conditions

Individuals with underlying risk factors such as obesity, cardiovascular diseases, diabetes mellitus, cancer, or inherited thrombophilia face heightened susceptibility to thrombosis after cyclones. These comorbidities may be poorly controlled due to disrupted medical care, further exacerbating thrombotic risk. Elderly populations and pregnant women also represent vulnerable groups with increased propensity for coagulopathy and venous thromboembolism^[^18^]^.

### Health care disruption and limited access to prophylaxis

Cyclones often cause significant damage to health care infrastructure, leading to shortages of diagnostic tools, anticoagulant medications, and trained personnel. Delays in recognizing and managing thrombotic complications increase morbidity. The inability to implement routine thromboprophylaxis in high-risk individuals due to resource constraints represents a critical gap in post-disaster care^[^6^]^.

## Clinical manifestations

The clinical presentation of thrombotic and coagulopathic complications in post-cyclone settings is often subtle at the outset, yet these conditions can rapidly progress to life-threatening outcomes if left unrecognized. Due to the chaotic and resource-limited nature of disaster environments, early symptoms may be overlooked or attributed to other causes. Clinicians and responders working in such settings must maintain a high index of suspicion, particularly among high-risk individuals^[^19^]^. DVT most commonly presents with unilateral swelling of the lower limb, often accompanied by pain, warmth, tenderness, and a sense of heaviness. The calf is frequently involved, although thigh veins may also be affected. The overlying skin may appear red or dusky, and superficial veins may become distended. In resource-poor settings, where Doppler ultrasonography may not be readily available, these signs become especially important for clinical diagnosis. However, DVT can remain clinically silent until complications such as PE occur^[^20^]^.

PE, a potentially fatal consequence of untreated DVT, may manifest with sudden onset of chest pain, shortness of breath, tachypnea, hemoptysis, or unexplained hypoxia. In some cases, PE may present atypically or mimic other conditions such as pneumonia, which is common in overcrowded shelters. A high clinical suspicion, particularly in individuals with known risk factors or recent immobility, is essential for timely diagnosis and treatment^[^21^]^. In addition to thrombotic events, coagulopathies such as DIC may occur, especially in the context of severe infection, trauma, or systemic inflammation. DIC presents with a paradoxical combination of thrombotic and hemorrhagic signs. Patients may exhibit signs of microvascular thrombosis – including organ dysfunction (e.g., renal failure, altered mental status) – as well as bleeding from mucosal surfaces, puncture sites, or into soft tissues. Petechiae, ecchymoses, hematuria, and gastrointestinal bleeding are common findings in overt DIC^[^22^]^.

Sepsis-associated coagulopathy, which may precede overt DIC, often presents with more subtle laboratory abnormalities such as mild thrombocytopenia, elevated D-dimer, and prolonged prothrombin time (PT). Clinically, these patients may appear septic with signs of systemic infection, fever, hypotension, and tachycardia, along with a gradual progression to multiorgan dysfunction. These signs should prompt early investigation and supportive care, particularly in environments prone to infectious disease outbreaks^[^23^]^. Superficial vein thrombosis (SVT), while less dangerous than DVT, can also occur and may present as a painful, firm, red cord-like structure beneath the skin, often in the lower extremities. Although traditionally considered benign, SVT may progress to DVT, especially when it involves proximal veins or occurs in high-risk individuals, and thus warrants clinical attention^[^24^]^. In pregnant and postpartum women, the clinical manifestations of thrombotic events can be masked by the normal physiological changes of pregnancy. Leg swelling, dyspnea, and fatigue are common in pregnancy but may also indicate an underlying DVT or PE. Vaginal bleeding or persistent abdominal pain in postpartum women may be signs of thromboembolic complications or coagulopathy and should be evaluated promptly^[^25^]^.

## Diagnostic considerations in disaster settings

Diagnosing thrombotic and coagulopathic complications in post-cyclone environments presents significant challenges due to resource limitations, disrupted health care systems, and the often-overwhelming burden of acute injuries and infectious diseases. In these settings, clinical acumen becomes a cornerstone of diagnosis, as access to confirmatory laboratory and imaging tools may be delayed or completely unavailable^[^26^]^. In the case of DVT, duplex Doppler ultrasonography remains the gold standard for diagnosis. However, in many disaster zones, this modality may not be accessible. In such instances, clinicians must rely heavily on clinical judgment supported by scoring systems such as the Wells Criteria, which assess risk based on symptoms like unilateral leg swelling, localized tenderness, recent immobilization, and history of previous DVT. A high clinical probability, especially in a high-risk environment, may justify empiric treatment when confirmatory testing is not possible^[^27^]^.

PE poses a greater diagnostic dilemma. While CT pulmonary angiography is the definitive tool for diagnosis, it is seldom available in emergency shelters or field hospitals. Pulse oximetry, although basic, can assist in identifying unexplained hypoxia, which may raise suspicion for PE. Clinical tools such as the revised Geneva score or Pulmonary Embolism Rule-out Criteria may offer structured guidance in triaging patients. In cases where diagnostic imaging is inaccessible, the presence of respiratory distress, chest pain, and tachycardia in a high-risk individual may warrant empirical anticoagulation under careful clinical monitoring^[^28^]^. Diagnosis of coagulopathy, particularly DIC, relies on a combination of laboratory markers including platelet count, PT, activated partial thromboplastin time (aPTT), fibrinogen levels, and D-dimer. In disaster settings, however, access to comprehensive coagulation panels is rare. Point-of-care testing (POCT), when available, can provide limited but valuable data such as INR or platelet count. In their absence, clinical indicators – such as mucosal bleeding, petechiae, spontaneous bruising, or signs of organ dysfunction – must be used as surrogate markers to raise suspicion for evolving DIC^[^29^]^.

Triage protocols in disaster scenarios should include simple screening tools to identify individuals at elevated risk of thrombotic events. Risk stratification based on age, immobility, comorbidities (e.g., cancer, pregnancy), and recent trauma or surgery can help prioritize limited diagnostic resources. For example, a bedridden elderly individual with leg swelling should be assessed early for DVT, even in the absence of imaging, and considered for prophylactic or therapeutic intervention based on risk-benefit analysis^[^30^]^. Where laboratory support is severely constrained, clinical gestalt and continuous monitoring gain importance. Serial physical examinations can help track the evolution of symptoms, while basic parameters such as heart rate, respiratory rate, temperature, and blood pressure may indirectly point toward underlying thromboembolic or septic processes. In cases of suspected DIC, close observation for evolving bleeding tendencies, reduced urine output, and altered mental status may offer critical diagnostic clues^[^31^]^. Importantly, documentation and referral systems – even if paper-based – should be maintained to facilitate continuity of care. When patients are transferred from field settings to tertiary hospitals, summary notes detailing suspected diagnoses, treatments initiated, and clinical progression are essential to avoid duplication or delay in care^[^32^]^.

## Prevention and management strategies

Effective prevention and management of DVT and coagulopathy in post-cyclone settings require a multifaceted approach that addresses the unique challenges posed by disaster environments. Proactive measures, timely diagnosis, and appropriate treatment are critical to reducing morbidity and mortality related to thrombotic complications^[^8^]^.

### Early mobilization

Encouraging movement and physical activity as soon as clinically feasible is one of the most important preventive strategies. Even simple leg exercises or periodic changes in position can enhance venous return and reduce venous stasis. In shelter settings, where space and resources may be limited, organized mobility programs or physical therapy interventions can help mitigate immobilization-related risks^[^33^]^.

### Hydration and nutritional support

Maintaining adequate hydration is essential to prevent hemoconcentration and hypercoagulability. Disaster response protocols should prioritize access to clean drinking water and electrolyte replacement for affected populations. Nutritional support that addresses deficiencies common in displaced individuals can also bolster overall vascular health and improve resistance to systemic inflammation and coagulopathy^[^34^]^.

### Pharmacological prophylaxis

For individuals identified as high risk – such as those with significant immobilization, prior history of thrombosis, or comorbidities like cancer or thrombophilia – pharmacological thromboprophylaxis should be considered. Low-molecular-weight heparin (LMWH) and other anticoagulants have proven efficacy in preventing DVT, but their use must be carefully balanced against bleeding risks, especially in settings where close monitoring is difficult. Protocols for safe administration and dose adjustment should be integrated into disaster medical plans^[^35^]^.

### Monitoring and early diagnosis

Rapid identification of DVT and coagulopathy is crucial to prevent complications like PE. Health care providers in disaster zones should be trained to recognize clinical signs such as limb swelling, pain, discoloration, and systemic symptoms suggestive of coagulopathy. Where possible, portable ultrasound devices and coagulation testing kits should be deployed to facilitate early diagnosis and guide management decisions^[^36^]^.

### Education and training

Capacity building among health care workers and disaster responders is vital. Training programs should emphasize risk assessment, prevention protocols, and recognition of thrombotic complications. Public awareness campaigns can also encourage affected individuals to remain mobile and hydrated and seek timely medical attention^[^37^]^.

### Health care infrastructure and resource allocation

Strengthening health care infrastructure resilience is a long-term goal that directly impacts thrombosis management post-disaster. Ensuring the availability of anticoagulant medications, diagnostic tools, and trained personnel must be prioritized in disaster preparedness and response plans. Coordination among governmental and non-governmental organizations can improve supply chains and facilitate continuity of care^[^6^]^.

### Integrated multidisciplinary care

Managing coagulopathy and DVT often requires a multidisciplinary approach involving emergency medicine, hematology, rehabilitation, and critical care teams. Early involvement of specialists can optimize treatment plans, including anticoagulation management, supportive care, and addressing underlying triggers such as infection or trauma^[^38^]^.

## Treatment considerations

Managing thrombotic and coagulopathic conditions in the aftermath of cyclone events poses unique challenges due to the disruption of health care infrastructure, limited availability of medications, and the high burden of acute illness and trauma. Treatment strategies must therefore balance clinical urgency with practical constraints, prioritizing interventions that are both evidence-based and feasible in low-resource settings^[^39^]^.

## Anticoagulation for thrombotic events

The cornerstone of DVT and PE management is anticoagulation. In disaster environments, LMWH is often the agent of choice due to its predictable pharmacokinetics, ease of subcutaneous administration, and lack of requirement for laboratory monitoring. LMWH is particularly advantageous where laboratory capacity is minimal or absent. In contrast, unfractionated heparin (UFH), while effective and less costly, requires frequent monitoring of activated partial thromboplastin time (aPTT), making it less suitable unless monitoring is available. Direct oral anticoagulants (DOACs) – such as rivaroxaban, apixaban, or dabigatran – may offer practical benefits, especially for ambulatory patients, due to fixed dosing and the absence of routine coagulation testing. However, their use may be constrained by cost, availability, and contraindications in patients with renal impairment, which can be undiagnosed in post-disaster settings. In some cases, warfarin may be used as a long-term solution, but it necessitates INR monitoring and dietary control, which are difficult to manage in the context of disrupted care^[^40^]^.

## Thrombolysis and surgical intervention

For massive PE or limb-threatening DVT with phlegmasia cerulea dolens, systemic thrombolysis or surgical thrombectomy may be indicated. However, these interventions are rarely feasible in cyclone-affected regions due to the need for intensive monitoring, surgical capacity, and specialized care. In most cases, treatment is limited to anticoagulation and supportive care, with stabilization and transfer to higher-level facilities when possible^[^41^]^.

## Management of coagulopathy and DIC

The treatment of coagulopathies, particularly DIC, hinges on identifying and addressing the underlying cause, such as infection, trauma, or obstetric complications. Supportive care is critical, including the administration of fresh frozen plasma, platelet transfusions, and cryoprecipitate as indicated, especially in patients with active bleeding or undergoing invasive procedures. In severely resource-limited settings, blood product availability may be scarce, necessitating the prioritization of life-threatening cases and the use of clinical judgment to guide transfusion decisions. Antibiotics and fluid resuscitation are crucial in cases of sepsis-associated DIC, and early empirical treatment may be life-saving in settings where laboratory confirmation is delayed. Close monitoring of vital signs and urine output, along with repeated clinical assessments, is essential in guiding ongoing management^[^42^]^.

## Special populations

In pregnant or postpartum women, the use of anticoagulants requires careful consideration. LMWH is generally preferred due to its safety profile during pregnancy and breastfeeding. However, in the absence of obstetric oversight, treatment must be guided cautiously, especially in the peripartum period where the risk of bleeding is high. For patients with renal impairment, dose adjustments for LMWH or DOACs may be necessary, but renal function may be difficult to assess in emergency settings, making UFH a safer choice if monitoring is available^[^43^]^.

## Continuity of care and follow-up

One of the greatest challenges in disaster scenarios is ensuring continuity of anticoagulation therapy. Displacement, medication shortages, and poor documentation increase the risk of missed doses, treatment interruption, or inappropriate dosing. Efforts should be made to establish mobile clinics, distribute educational materials, and maintain simple medication logs or identification cards for individuals on anticoagulation. Community health workers can play a vital role in ensuring follow-up and adherence, particularly once the acute phase of the disaster has passed^[^44^]^.

## Infection control and supportive measures

Since infections can exacerbate both thrombosis and coagulopathy, infection control – including timely antibiotic use, wound care, and vaccination – is integral to treatment. Hydration, mobility support, and nutritional supplementation also serve as important supportive measures that can reduce the progression of thrombotic risk and improve recovery^[^45–48^]^.

## Conclusion

DVT and coagulopathy represent significant, yet often underrecognized, health challenges in the aftermath of cyclone events. The unique combination of prolonged immobilization, dehydration, trauma, systemic inflammation, and disrupted health care services creates an environment highly conducive to thrombotic complications. Understanding the complex pathophysiological mechanisms and identifying risk factors specific to post-cyclone settings are essential steps toward effective prevention and management. Implementing early mobilization, ensuring adequate hydration, and providing targeted pharmacological prophylaxis where feasible are key strategies to mitigate the risk of thrombosis and coagulation disorders in affected populations. Strengthening health care infrastructure, enhancing provider education, and integrating thrombosis risk assessment into disaster response protocols further improve clinical outcomes. Continued research is needed to optimize diagnostic tools and treatment approaches tailored to resource-limited and high-stress disaster environments.

## Data Availability

The data that support the findings of this study are available from the corresponding author upon reasonable request.
